# First person – David Pirovich

**DOI:** 10.1242/bio.051631

**Published:** 2020-03-24

## Abstract

First Person is a series of interviews with the first authors of a selection of papers published in Biology Open (BiO), helping early-career researchers promote themselves alongside their papers. David Pirovich is first author on ‘*Schistosoma mansoni* glyceraldehyde-3-phosphate dehydrogenase enhances formation of the blood-clot lysis protein plasmin’, published in BiO. David is a PhD candidate in the lab of Dr Patrick Skelly at Tufts University, North Grafton, MA, USA, investigating novel moonlighting functions (thrombolysis, immunomodulation) of *Schistosoma mansoni* glycolytic enzymes.


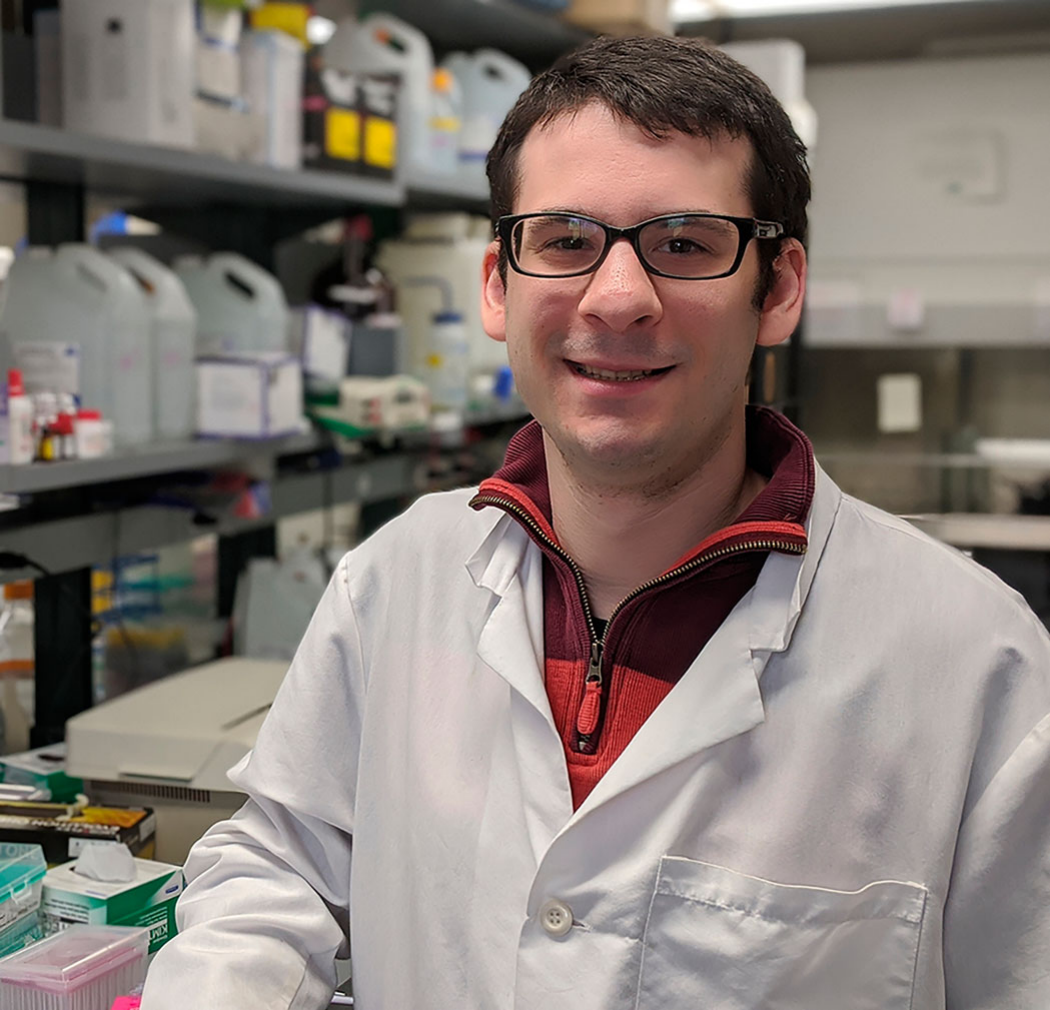


**David Pirovich**

**What is your scientific background and the general focus of your lab?**

I graduated from the School of Environmental and Biological Sciences at Rutgers University with a degree in microbiology. After working in a cancer research lab for 2 years, I joined the Molecular Helminthology Laboratory at the Cummings School of Veterinary Medicine at Tufts University to fulfil my goals of working with parasites in a laboratory setting and contributing to communicating the importance of neglected tropical diseases to the scientific community.

Our laboratory is interested in host-parasite interactions between the parasitic flatworm *S. mansoni* and its human hosts. To elucidate this relationship, we study both the characterization of the molecular components of the tegument (skin) of the parasitic *S. mansoni* blood fluke and how these components of parasite tegument interact with host factors in order to promote survival *in vivo*.

**How would you explain the main findings of your paper to non-scientific family and friends?**

All living organisms use a series of biochemical reactions to generate energy. One of these energy-generating processes is called glycolysis, and it is a ten-step pathway with a series of enzymes facilitating each step along the way. Interestingly, some of these enzymes have been detected on the outer surface (called the tegument) of a parasitic worm called *S. mansoni*. Previous research indicates that pathogenic organisms sometimes utilize these surface-bound enzymes for additional, or ‘moonlighting’, functions, which assist in their survival. We have found that one of these surface-bound enzymes (GAPDH) is indeed present on the surface of the *S. mansoni* worms, and it can activate a blood-clotting enzyme (called plasmin) in a manner that may allow it to facilitate the breakdown of blood clots.

“[…] the parasites are equipped with an additional surface-bound glycolytic enzyme (GAPDH) serving a novel moonlighting function, which facilitates the conversion of plasminogen to plasmin, a fibrinolytic enzyme.”

**What are the potential implications of these results for your field of research?**

The results of our paper suggest an additional mechanism that allows the *S. mansoni* worms to survive within their hosts for up to a decade; armed with surface-bound glycolytic enzymes such as enolase (which has previously been described by our lab as a plasminogen-activating enzyme), our work here shows that the parasites are equipped with an additional surface-bound glycolytic enzyme (GAPDH) serving a novel moonlighting function, which facilitates the conversion of plasminogen to plasmin, a fibrinolytic enzyme. Equipping themselves with enzymes that can facilitate fibrinolysis, the worms can essentially degrade blood clots that begin to form around them within the vascular microenvironment, and this may also allow the worms better movement and survival within their hosts’ blood vessels. Furthermore, the results provide further insight into utilizing GAPDH as a potential vaccine or drug target, as localizing the enzyme to the surface of the worms reveals an accessible site for therapeutic agents to target.

**What has surprised you the most while conducting your research?**

I was quite surprised to see that performing gene knockdown using small interfering RNA (siRNA) did not significantly impact the viability of the worms, which were electroporated with an siRNA targeting GAPDH. As this is a ubiquitous housekeeping gene that is crucial to parasite survival, it was surprising to see that suppression of such a vital gene not only lowered gene and protein expression of this essential enzyme, but the worms themselves survived and showed the same viability as the worms that were given control or no siRNA treatments. It is possible that these worms generate so much GAPDH at a rapid rate that the gene/protein reduction induced by RNA interference (RNAi) does little to affect the capability of the functional GAPDH that remains, or that these worms can revert to alternative methods of energy production during times of glycolytic stress.

**What, in your opinion, are some of the greatest achievements in your field and how has this influenced your research?**

I believe in the field of parasitology, the ability to suppress or even totally knock out genes using RNAi and CRISPR has major implications for the field in that generating specific mutant strains of parasites allows researchers a more refined measure of observing minute differences in parasite viability and virulence in modeling parasitic disease. RNAi has allowed me to quantify whether surface GAPDH activity is reduced when schistosome parasites are electroporated with an siRNA targeting GAPDH, which helped confirm the presence of the enzyme at the parasite surface in addition to immunostaining. Furthermore, the ability to use harmless bacteria to express parasite recombinant proteins has allowed countless scientists to study enzymes without exposure to potentially harmful pathogens, and has allowed me to characterize recombinant *S. mansoni* on molecular level and get a better understanding of the conditions this enzyme needs to function at an optimal level.

**Figure:**
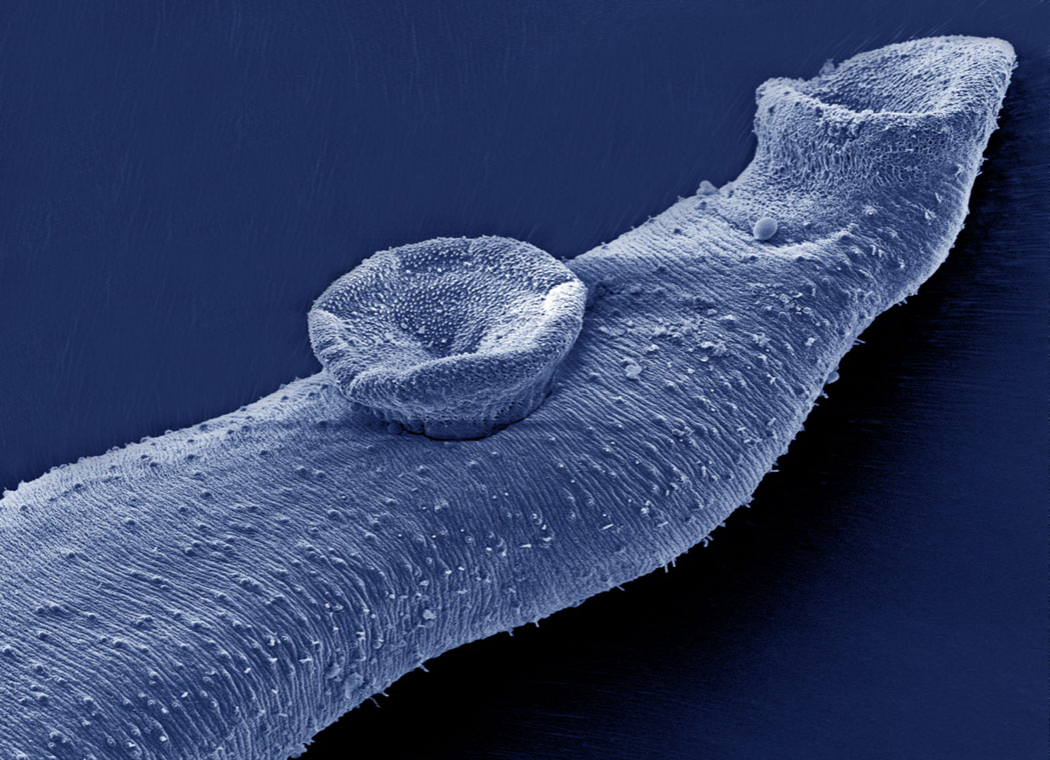
**A scanning electron micrograph of the anterior end of an adult male *Schistosoma mansoni* parasite.**

“[…] there should be additional focus in universities to teach early-career scientists how to effectively write grants, especially those who are thinking of running a lab in the future.”

**What changes do you think could improve the professional lives of early-career scientists?**

I think that apart from learning appropriate lab and presentation skills, and how to conduct research appropriately, there should be additional focus in universities to teach early-career scientists how to effectively write grants, especially those who are thinking of running a lab in the future. This would give early-career scientists a clearer picture about the inner workings of securing funding for the lab, as well as communicating to a panel of researchers the most pertinent information to earn said funding. Furthermore, there should be an outlet (such as a newsletter) organized by government-funded scientific organizations, e.g. the National Institutes of Health, for PhD students to effectively communicate to both the scientific community and the public the basis of their work to sate growing concerns about scientific misconduct.

**What's next for you?**

I am continuing research to fulfil my thesis requirement to graduate with a PhD, then I will hopefully move into the research/industry sector to work as a full-time scientist. I am also interested in joining a publication company to work as an editor. I am keeping the door open to moving into a postdoctoral position as well. I would also like to expand into science writing or podcasting in order to better broadcast the importance of understanding and funding neglected tropical diseases to the public.
